# Small-angle X-ray scattering characterization of a $\beta$-amyloid model in phantoms

**DOI:** 10.1186/s13104-020-04969-8

**Published:** 2020-03-04

**Authors:** Sophya Breedlove, Jasson Crentsil, Eshan Dahal, Aldo Badano

**Affiliations:** 1 Division of Imaging, Diagnostics, and Software Reliability, Office of Science and Engineering Laboratories, Center for Devices and Radiological Health, Food and Drug Administration, Silver Spring, MD USA; 2 Fischell Department of Bioengineering, University of Maryland, College Park, MD USA; 3 Department of Materials Science and Engineering, Carnegie Mellon University, Pittsburgh, PA USA; 4 Department of Chemical, Biochemical and Environmental Engineering, University of Maryland Baltimore County, Baltimore, MD USA

**Keywords:** Amyloid, SAXS, Alzheimer’s disease

## Abstract

**Objective:**

We present a method to prepare an amyloid model at scalable quantities for phantom studies to evaluate small-angle x-ray scattering systems for amyloid detection. Two amyloid models were made from a plasma protein with and without heating. Both models mimic the $\beta$-sheet structure of the $\beta$-amyloid ($\beta \text {A}$) plaques in Alzheimer’s disease. Amyloid detection is based on the distinct peaks in the scattering signature of the $\beta$-sheet structure. We characterized the amyloid models using a spectral small-angle x-ray scattering (sSAXS) prototype with samples in a plastic syringe and within a cylindrical polymethyl methacrylate (PMMA) phantom.

**Results:**

sSAXS data show that we can detect the scattering peaks characteristic of amyloid $\beta$-sheet structure in both models around 6 and 13 $\text {nm}^{-1}$. The $\beta \text {A}$ model prepared without heating provides a stronger signal in the PMMA phantom. The methods described can be used to prepare models in sufficiently large quantities and used in samples with different packing density to assess the performance of $\beta \text {A}$ quantification systems.

## Introduction

Non-invasive detection of $\beta$-amyloid ($\beta \text {A}$) plaques could facilitate the diagnosis and treatment of neurodegenerative diseases such as Alzheimer’s disease (AD). Currently, $\beta \text {A}$ plaques cannot be detected antemortem without a contrast agent. In order to detect $\beta \text {A}$ plaques in a label-free way, a technique may be employed that identifies the plaques based on unique structural features. In this context, the amyloid model used to evaluate this detection technique must contain the same characteristic structural features of human $\beta \text {A}$ plaques.

Aggregation of $\beta \text {A}$ peptides into fibrils leads to the formation of senile plaques. Such $\beta \text {A}$ plaques are rich in $\beta$-sheets and are characterized by a cross-$\beta$ structure, where the $\beta$-sheets run parallel to the fibril axis and the constitutive $\beta$-strands run perpendicular [1–3]. This distinctive cross-$\beta$ structure results in a unique X-ray diffraction pattern, with a peak at 4.7 Å, corresponding to $\beta$-strand spacing, and a peak at 10 Å, corresponding to $\beta$-sheet spacing [3]. Bovine serum albumin (BSA) is a readily available plasma protein that can undergo similar aggregation under laboratory conditions forming higher-order fibril structures with cross-$\beta$ sheets. Upon heating a BSA solution, BSA is reported to form an elongated fibril structure with an extensive $\beta$-sheet character that binds to amyloid-specific dyes [4], making it an ideal amyloid model. $\beta$-sheet content in BSA has been found to increase up to $40\%$ upon thermal aggregation [5]. This would facilitate phantom studies where modulation of the $\beta$-sheet signal is required.

Another consideration for studying an amyloid model in phantoms is the performance of the detection technique through thick samples. In order to non-invasively detect human $\beta \text {A}$ plaques antemortem, the detection method must identify $\beta$-sheet character through an object the size of a human head. Previously, synchrotron-based Fourier transform infrared micro-spectroscopy is successfully used to detect $\beta$-sheet content increase in the brain tissue of transgenic AD mice [6]. Human $\beta \text {A}$ plaques have also been studied in thin sections of human brain using X-ray diffraction (XRD) [7]. The low energy and limited angular range in X-ray diffraction make it difficult to study thick samples. Therefore, in this study, we use spectral small-angle X-ray scattering (sSAXS) to characterize our amyloid models. sSAXS is a technique that collects SAXS data using high energy polychromatic X rays and a spectroscopic detector. sSAXS is suitable to study both thick and thin samples as the energy range can be chosen to maximize coherent scattering per deposited energy. Recently, Choi et al. simulated a SAXS-CT system to image amyloid plaques in small animals and humans [8].

In this work, we present two practical methods for preparing an amyloid model, with and without heating, that mimics the $\beta$-sheet structure characteristic of the human $\beta \text {A}$ plaques associated with AD. These models are used in syringe and phantom studies to evaluate a sSAXS prototype ability to detect the $\beta$-sheet structure of an amyloid model without a contrast agent.

## Main text

## Methods

### Amyloid model preparation

**Fig. 1:**
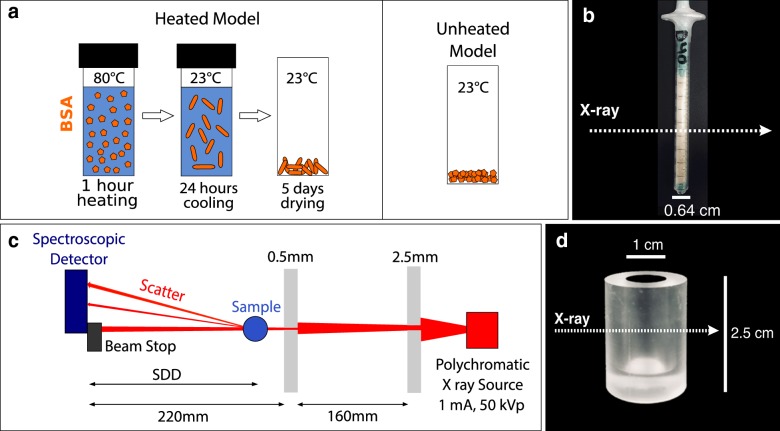
Model preparation and experimental setup. **a** Schematic of $\beta \text {A}$ model preparation, heated (left) and unheated (right). **b** Syringe sample of an amyloid model. **c** sSAXS system setup. **d** Dimensions of the cylindrical PMMA phantom

As illustrated in Fig. 1a, we prepared two different amyloid models from bovine serum albumin (BSA) powder (Sigma Aldrich A7030-100G), one with heating and another without heating.

To prepare the unheated model, the lyophilized powder of BSA was crushed into a fine-grained powder and packed into either a plastic syringe or a cylindrical phantom. To prepare the heated model, BSA was first dissolved in aqueous buffer (10 mM phosphate, 150 mM NaCl, pH 7.4) to make 20, 30, and 40 mg/ml of BSA solutions. Each BSA solution was heated in a water bath for 1 h at 80 ± 1.5 °C followed by cooling the solution/gel at room temperature for 24 h and drying for 5 days. The dried samples were then crushed into powder to fill syringes for SAXS measurements (Fig. 1b). The syringe was 6.4 mm thick. The packing density of the BSA powder was around 677 $\text {mg/cm}^{3}$.

### Data collection and processing

All SAXS measurements were acquired using the spectral SAXS (sSAXS) setup presented in Fig. 1c. A polychromatic X-ray beam from a tungsten source (50 kVp and 1 mA) was collimated by two lead pinholes of 2.5 and 0.5 mm diameters. An 80 $\times$ 80 pixels HEXITEC detector (Quantum Detectors Ltd) made from cadmium telluride crystal was used to measure the position and energy of the scattered photons in each pixel. The sample to detector distance (SDD) was 214 mm. Data were acquired for 600 and 1800 s for the syringe study and 1200 s for the phantom study. Data were analyzed in terms of the momentum transfer *q*. *q* is related to energy (*E*) and scattering angle $(2\theta )$ by $q = 4\pi E \sin \theta / hc$, where $hc = 1.24$ keV nm. The accessible q-range was from 1.3 to 28 $\text {nm}^{-1}$.

Q-data from 30 to 45 keV was summed for all energy bins to obtain counts as a function of q, *C*(*q*). The binning step was 1.2 $\text {nm}^{-1}$. The background correction was done on the summed q-data to remove background scattering. The characteristic Bragg peaks of caffeine powder were used to calibrate the sSAXS system and validate the data analysis process. A hollow cylindrical phantom was used to assess the capability of the sSAXS system to detect amyloid targets inside phantoms. The cylindrical phantom was made from polymethyl methacrylate (PMMA) with a 1 cm internal diameter and 2.5 cm height (Fig. 1d).

**Fig. 2:**
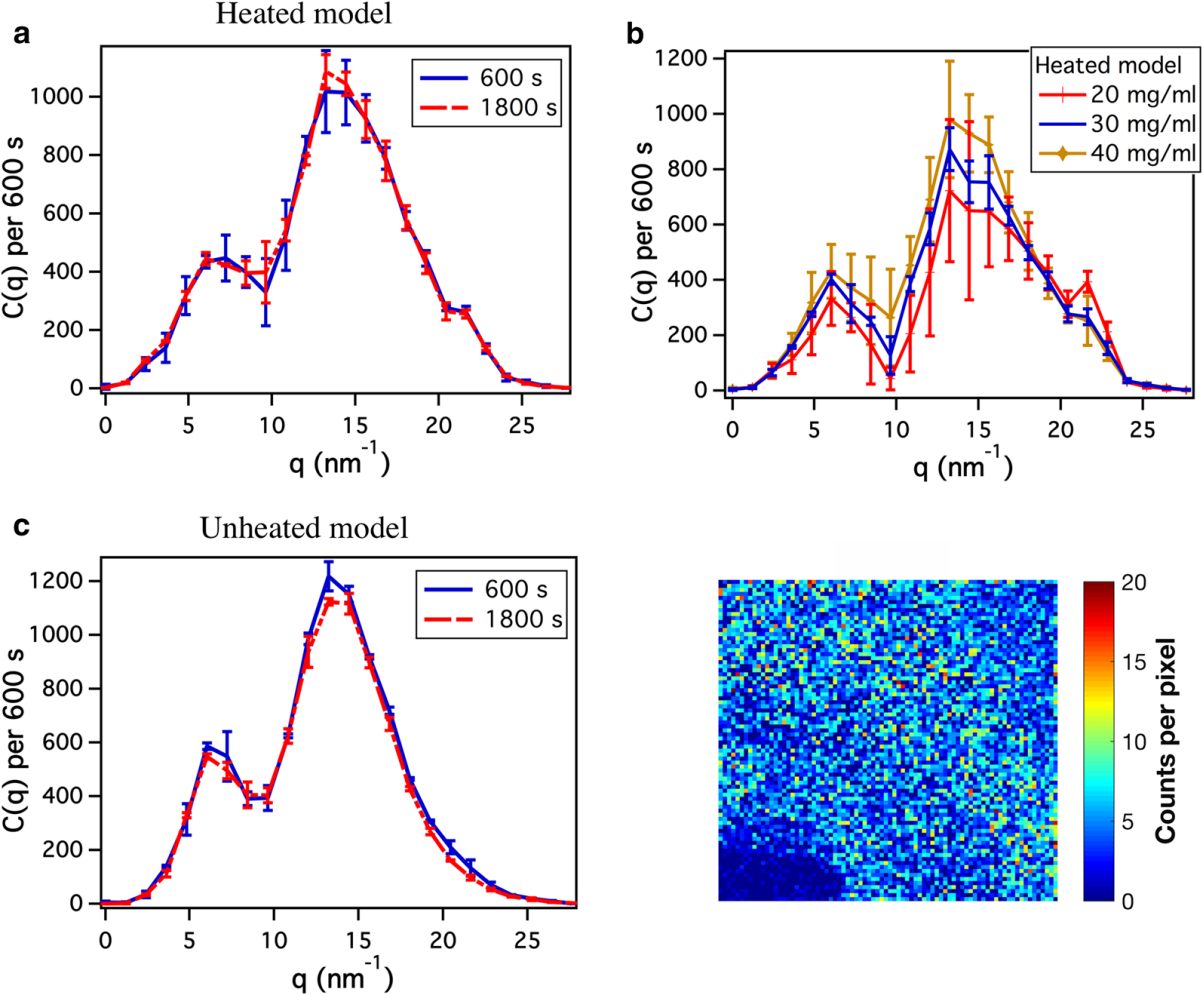
Characterization of the heated and unheated $\beta \text {A}$ models in a syringe. **a** Scattering signal of the heated model using 600 and 1800 s data acquisition times. **b** Effect of BSA concentration in the scattering signal of the heated model. Error bar represents standard deviations from $\text {n}=3$ measurements. **c** Scattering signal of the unheated model for 600 and 1800 s from $\text {n}=2$ measurements. **d** Representative 2D detector data of the unheated model in 30 to 45 keV energy range

**Fig. 3:**
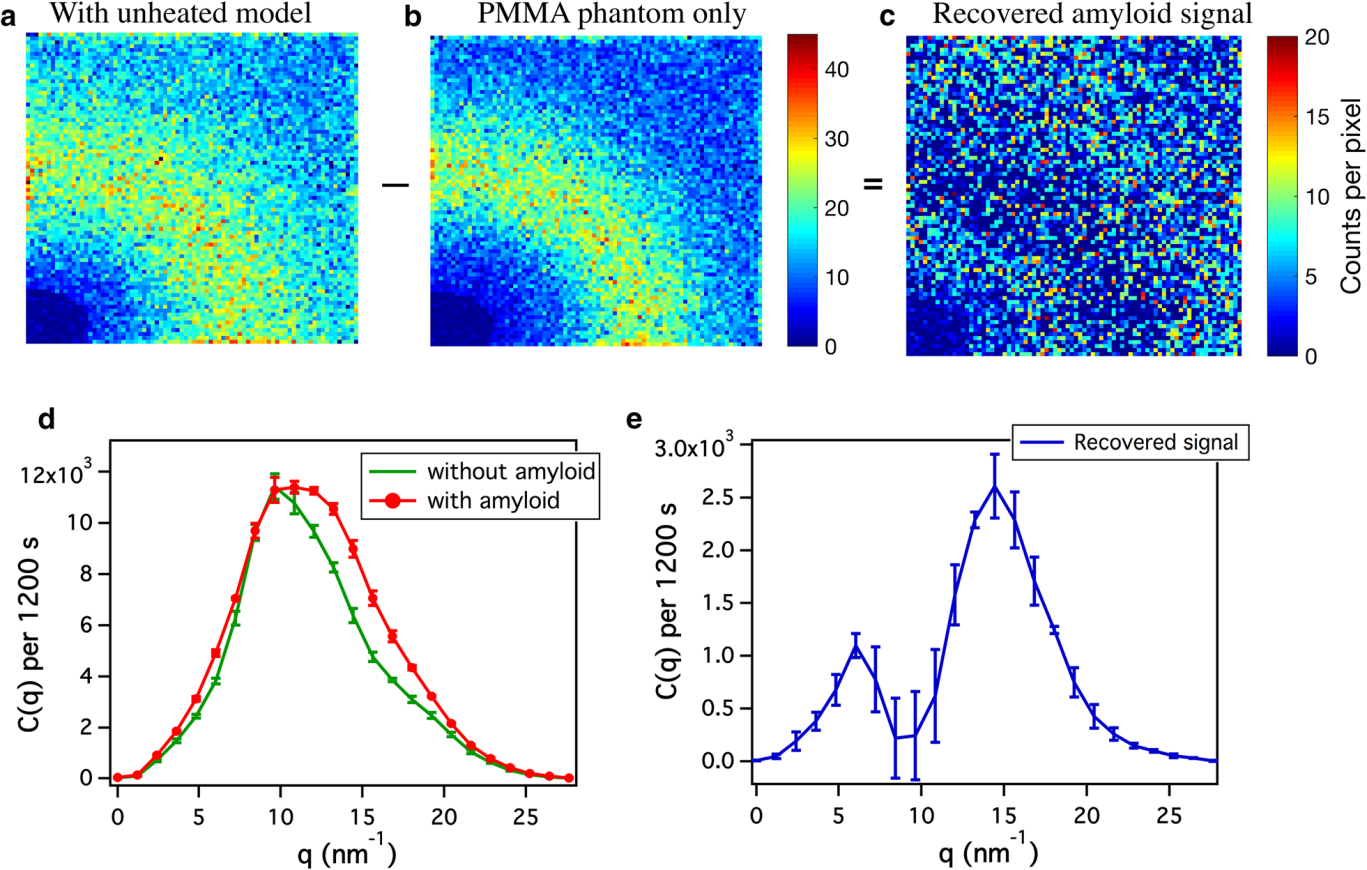
Characterization of the unheated $\beta \text {A}$ model in a cylindrical PMMA phantom. **a**–**c** 2D detector data before and after background subtraction. **d** Scattering signal from the PMMA phantom with and without the unheated $\beta \text {A}$ model. **e** Recovered scattering peaks associated with amyloids after background subtraction. Error bar represents standard deviations from n = 3 measurements

## Results

### Syringe samples

Results of the syringe study for both the heated and unheated $\beta \text {A}$ models show easily distinguishable scattering peaks around 6 and 13 nm^−1^ (Fig. 2), corresponding to the characteristic peaks of $\beta \text {A}$ plaques or fibrils resulting from their cross-$\beta$ structure [7, 9]. The peak around 6 nm^−1^ corresponds to the $\beta$-sheet spacing and the peak around 13 nm^−1^ corresponds to the $\beta$-strand spacing. 600 s data acquisition time was sufficient to identify these peaks in both models. In the heated amyloid model, the initial BSA solution concentration can be changed to increase or decrease the strength of the scattering peaks from $\beta$-sheets (Fig. 2b). However, there is high uncertainty in measuring those peaks for the 20 mg/ml sample. The unheated $\beta \text {A}$ model showed slightly sharper peaks around 6 and 13 nm^−1^ (Fig. 2c). Its background-corrected 2D scattering pattern is shown in Fig. 2d.

### Phantom samples

We then used sSAXS to characterize our $\beta \text {A}$ models in the 2 cm thick PMMA phantom. This is relevant for the purpose of evaluating the performance of the detection technique through thick samples. We found that the heated model did not provide sufficient $\beta$-sheet signal to be reliably used in the phantom study. Therefore, Fig. 3 shows the results only for the phantom with an unheated $\beta \text {A}$ model. The unheated model provided sufficient scattering signal after background correction (Fig. 3c), and peaks were identifiable around 6 and 14 nm^−1^ (Fig. 3e). It should be noted that the peaks were recovered without accounting for the attenuation effect. Also, as reported before [10], PMMA alone has a strong scattering peak around 9.64 nm^−1^ (Fig. 3d).

## Discussion

Both the heated and unheated $\beta \text {A}$ models mimic the cross-$\beta$ structure of $\beta \text {A}$ plaques. The heated $\beta \text {A}$ model is primarily useful in estimation tasks in thin (< 1 cm) tissue-mimicking phantoms. For instance, using a heated $\beta \text {A}$ model of various concentrations, an X-ray scattering system can be calibrated for estimating amyloid plaque density in a 5 mm thin phantom representing a brain tissue slice. High concentration of BSA (> 20 mg/ml) was required to prepare the heated $\beta \text {A}$ model in order to decrease the uncertainty in measuring the characteristic $\beta$-sheet peaks. The heated BSA solution at high concentration forms $\beta$-sheet rich structures in the gel state. Mechanistic aspects of BSA aggregation and gelation are well-studied [11, 12].

The unheated $\beta \text {A}$ model, on the other hand, is useful for evaluating and calibrating an X-ray scattering prototype when detecting $\beta$-sheet structure through thick phantoms beyond 1 cm. The unheated model represents amorphous aggregates of BSA and has sharper and more detectable $\beta$-sheet scattering signal through thick phantoms. The beta-sheet content in the lyophilized BSA is approximately 25% [13]. Two characteristic peaks of the $\beta$-sheets were recovered in our phantom study despite the strong scattering from PMMA (see Fig. 3b). However, the second peak of $\beta$-sheet at 13 nm^−1^ was found to be around 14 nm^−1^. This slight shift in the peak position is not significant considering the q-binning step of 1.2 nm^−1^.

Both heated and unheated $\beta \text {A}$ models are useful alternatives to testing with human amyloid protein aggregates, since they mimic the $\beta$-sheet structure associated with $\beta \text {A}$ fibrils, and they can be inexpensively (approx. $\$0.011$ per mg) produced in large quantities.

## Limitations

- No correction for the attenuation effect in the samples due to self-absorption of X-ray quanta.
- High packing density of BSA used compared to realistic levels in early AD brains.
- Exact percentage of $\beta$-sheet content is not characterized.

## Data Availability

The datasets used and/or analysed during the current study are available from the corresponding author on reasonable request.

## Abbreviations

$$\beta \text {A}$$: $$\beta $$-Amyloid
AD: Alzheimer’s disease
sSAXS: Spectral small-angle x-ray scattering
SAXS: Small-angle X-ray scattering
BSA: Bovine serum albumin
PMMA: Polymethyl methacrylate
SDD: Sample to detector distance
